# Characterization of CYP71AX36 from Sunflower (*Helianthus annuus* L., Asteraceae)

**DOI:** 10.1038/s41598-019-50520-6

**Published:** 2019-10-04

**Authors:** Maximilian Frey, Iris Klaiber, Jürgen Conrad, Aylin Bersch, Irini Pateraki, Dae-Kyun Ro, Otmar Spring

**Affiliations:** 1 Institute of Botany, University of Hohenheim, Garbenstraße 30, 70593 Stuttgart, Germany; 2 Mass Spectrometry Unit, Core Facility Hohenheim, University of Hohenheim, Emil-Wolff-Str. 12, 70599 Stuttgart, Germany; 3 Institute of Chemistry, University of Hohenheim, Garbenstraße 30, 70593 Stuttgart, Germany; 4 Department of Plant and Environmental Sciences, Faculty of Science, University of Copenhagen, Thorvaldsensvej 40, Frederiksberg C, Denmark; 5 Department of Biological Sciences, University of Calgary, Calgary, T2N 1N4 Canada

**Keywords:** Oxidoreductases, Molecular engineering in plants, Enzymes, Biosynthesis

## Abstract

Sesquiterpene lactones (STL) are a subclass of isoprenoids with many known bioactivities frequently found in the Asteraceae family. In recent years, remarkable progress has been made regarding the biochemistry of STL, and today the biosynthetic pathway of the core backbones of many STLs has been elucidated. Consequently, the focus has shifted to the discovery of the decorating enzymes that can modify the core skeleton with functional hydroxy groups. Using *in vivo* pathway reconstruction assays in heterologous organisms such as *Saccharomyces cerevisiae* and *Nicotiana benthamiana*, we have analyzed several cytochrome P450 enzyme genes of the CYP71AX subfamily from *Helianthus annuus* clustered in close proximity to one another on the sunflower genome. We show that one member of this subfamily, CYP71AX36, can catalyze the conversion of costunolide to 14-hydroxycostunolide. The catalytic activity of CYP71AX36 may be of use for the chemoenzymatic production of antileukemic 14-hydroxycostunolide derivatives and other STLs of pharmaceutical interest. We also describe the full 2D-NMR assignment of 14-hydroxycostunolide and provide all 13C chemical shifts of the carbon skeleton for the first time.

## Introduction

**Figure 1:**
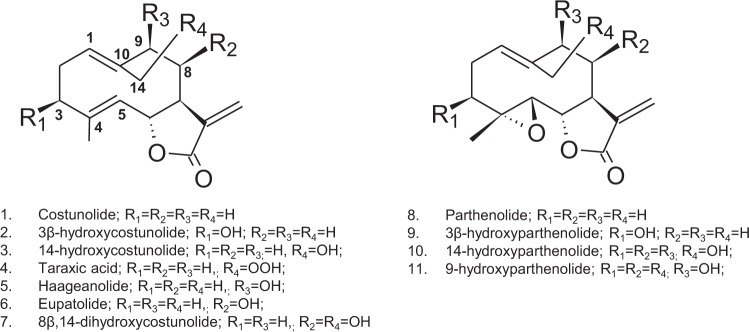
Derivatives of costunolide. A selection of costunolide derivatives found in *Asteraceae* plant species and biotechnologically produced by expression of cytochrome P450 enzymes with hydroxylations at positions C3, C8, C9 and C14 and epoxidation at C4-C5. Note: The 1(10) double bond is E for compounds 1, 2, 5, 6, 8, 9 and 11. Due to a change in substituent priority in compound 3, 4, 7 and 10 the 1(10) double would be defined as Z according to IUPAC nomenclature, however, in the literature the germacranolide backbone is predominantly defined as a 1(10)4(5)-(E,E)diene, regardless of the substituent.

Sesquiterpene lactones (STL) are a diverse group of specialized plant metabolites, particularly characteristic to the Asteraceae family, which contain a C15 backbone and an α-methylene γ-lactone ring^1^. Recently, the enzymatic steps leading to the basic 6,7-*cis* and 7,8-*trans* STL structures, such as costunolide (**1**) and inunolide, have been described. In this context, Liu *et al*.^2^ discovered the enzyme TpPTS/CYP71CA1 which can convert costunolide (**1**) to parthenolide (**8**) and Frey *et al*.^3^ found the biosynthetic pathway for the formation of eupatolide (8β-hydroxylated costunolide, (**6**)) from costunolide (Fig. 1). Thus, pathway reconstructions in heterologous hosts have confidently characterized the biosynthetic steps necessary for the biosynthesis of a set of basic STL core skeletons, which can serve as lead structures of many pharmaceutically interesting compounds. At the same time, diverse costunolide derivatives in the Asteraceae family show that this plant family holds the catalytic potency to introduce many stereospecific hydroxylations^4^. Liu *et al*.^2^ also discovered the enzyme Tp8878/CYP71CB1 which can introduce a 3β-group to either costunolide (**1**) or parthenolide (**8**), leading to 3β-hydroxycostunolide (**2**) or 3β-hydroxyparthenolide (**9**), respectively. With the basic pathway to STL core skeletons being identified, it is possible to characterize the next steps towards the decorations of the above-mentioned core skeletons. The introduction of a 14-hydroxy-group on the costunolide skeleton for example, can lead to the synthesis of several compounds with demonstrated pharmacological activities, such as 8β,14-dihydroxycostunolide (**7**)^5^, taraxinic acid (**4**)^6^ and 14-hydroxyparthenolide (**10**)^7^. Tyagi *et al*.^7^ converted parthenolide to 9β-hydroxyparthenolide (**11**) and 14-hydroxyparthenolide, (**10**) by modifying the bacterial enzyme CYP102A1, allowing the introduction of side chains on the core structure to improve the water solubility without reducing the bioactivity of parthenolide.

Recently, cytochrome P450 enzymes from several metabolic pathways have been reported to be arranged in gene clusters^8^. For cytochrome P450 enzymes in sesquiterpenoid pathways of Asteraceae, similar substrates and reaction types appear to be linked to specific subfamilies, such as the multi-step oxidation of un-oxidized sesquiterpenes in the CYP71AV1 subfamily or the use of germacrene A acid as a substrate in the CYP71BL subfamily^3^. In this paper we present the elucidation of cytochrome CYP71AX36 a 14-hydroxylase from *Helianthus annuus* in yeast and *Nicotiana benthamiana*, that is co-localized with other CYP71AX genes in a gene cluster on chromosome five.

## Results and Discussion

### CYP71AX36 converts costunolide to 14-hydroxycostunolide in yeast

**Figure 2:**
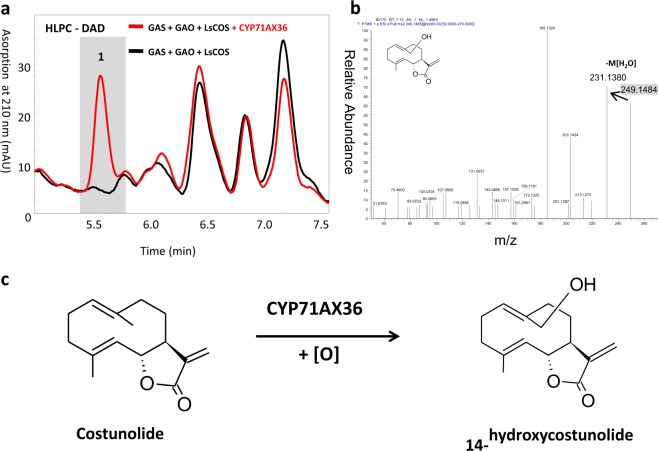
Characterization of CYP71AX36 yeast. Comparison of yeast strain [EPY300-Leu2d-GAS/CR/GAO/LsCOS|Ura- CYP71AX36] (n = 4) with control [EPY300-Leu2d-GAS/CR/GAO/LsCOS|Ura-empty] (n = 4). (**a**) DAD chromatogram, with peak a at 5.58 min. (**b**) MS^2^ spectrum of peak a. (**c**) Reaction catalyzed by CYP71AX36.

The cDNAs of nine cytochrome P450 candidate genes previously identified in capitate glandular trichome transcripts^3,9^ were cloned into yeast expression vectors as previously described^3^. The well-established yeast expression system using the FPP-overproducing yeast strain EPY300^9,10^ was used to assess the catalytic activity of these 9 candidate P450 cDNAs. In EPY300 background, the cDNAs responsible for the biosynthesis of STL skeletons (germacrene A acid, 8β-hydroxygermacrene A acid or costunolide) cloned in the vector pESC-Leu2d were co-expressed together with individual CYP candidate cDNA cloned in the vector pESC-Ura. This system supplies substrates *in vivo* and enabled us to examine the catalytic activities of the selected CYP enzymes (Supplementary Fig. S1). All candidates were tested for oxygenation activities of germacrene A acid, 8β-hydroxy-germacrene A acid and costunolide as previously described^3^. With the exception of CYP71AX36, no new enzyme product was detected. Expression of *CYP71AX36* in the yeast strain producing costunolide (EPY300-Leu2d-GAS/GAO/CR/LsCOS|Ura-CYP71AX36) resulted in a new peak (peak 1) on the HPLC chromatogram in comparison to the control experiment (EPY300-Leu2d-GAS/GAO/CR/LsCOS|Ura-empty) (Fig. 2a). Peak 1 had a retention time of 5.6 min and a λ_max_ of 210 nm with a UV-Vis spectrum, typical for STLs. LC-MS analysis of this compound showed a mass of [M + H]^+^ = 249.2, corresponding to the mass of mono-hydroxylated costunolide (C_15_H_20_O_3_) (Fig. 2b). A fragment of [M + H − H_2_O]^+^  = 231.3, resulting from the loss of one water molecule, further corroborated that a single oxygen was incorporated to the costunolide backbone.

**Figure 3:**
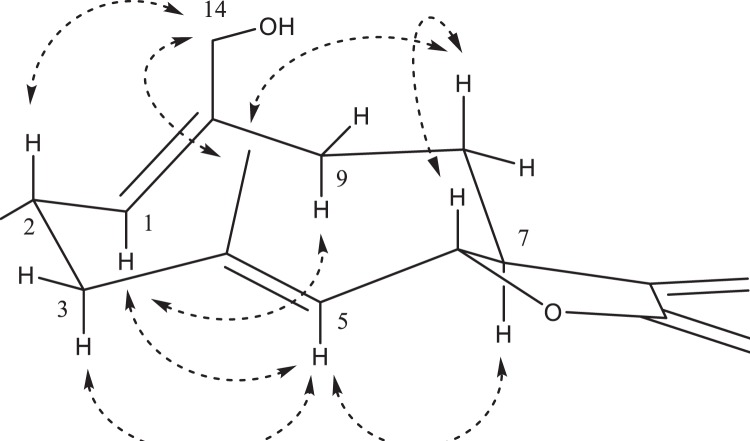
3D structure of 14-hydroxycostunolide derived by extensive NMR analysis. Important ROESY correlations are shown by arrows.

The observed shift in the retention time of ΔRT = 9.6 min inducted by this oxidation of costunolide (15.5 min) was stronger compared to other mono-hydroxylated costunolide derivatives available as reference compounds (8β-hydroxylation: 6.1 min, 9β-hydroxylation: 6.1 min, 15-hydroxylation: 8.0 min). We therefore assumed that another and more exposed position in the costunolide skeleton was hydroxylated that induced a higher shift in retention time. Upscaling the yeast culture to 0.5 liter enabled us to purify a sufficient amount of this novel product to conduct extensive NMR analyses. The structure of this compound was unambiguously identified as 14-hydroxycostunolide by evaluations of all 1D and 2D homo- and heteronuclear NMR spectra (Table 1, Supplementary Figs S1, S3–S7). The relative stereochemistry as shown in Fig. 3 and Supplementary Fig. S2 was derived by ROESY. A ROE between H-5 at *δ* 4.79 ppm and H-3ax at *δ* 2.10 ppm indicated an E 4(5) double-bond whereas ROESY correlations between H-2ax at *δ* 2.34 ppm and H-14 at *δ* 3.79 ppm as well as H-1 at *δ* 5.00 ppm and H-9ax at *δ* 2.01 ppm established a Z 1(10) double-bond. The partially assigned ^1^H NMR data reported in the literature^11^ are in good accordance with our findings. The modeling of 14-hydroxycostunolide (Fig. 3, Supplementary Fig. S2) showed that the hydroxy-group at C14 is more exposed to solvent than those at other positions in the costunolide backbone, providing an explanation as to why the hydroxylation at C14 induces a higher shift in retention time than other hydroxy costunolides (e.g., C8 and C9 hydroxycostunolides).

**Table 1:** NMR Data of 14-hydroxycostunolide, purified from yeast

Position	^13^C 125 MHz	^1^H 500 MHz
	(δ, ppm)	(δ, mult; J[Hz])
1	130.5	5.00, dd; 5.1, 12.3
2	25.4	2.23, eq, m 2.34, ax, dddd, 4.9, 3 × 12.5
3	39.0	2.32, eq, ov 2.10, ax, ddd; 5.6, 2 × 13.5
4	141.0	
5	127.2	4.79, bd; 10.0
6	81.6	4.54, ax, dd; 8.7, 9.9
7	50.2	2.59, ax, dtt-*like*; 1.2, 2 × 3.4, 2 × 9.3
8	28.3	1.73, ax, dddd, 2.0, 9.4, 12.1, 14.5
9	35.9	2.91,eq, bdd; 6.4, 13.6 2.01, ax. bdd, 2 × 12.5
10	139.5	
11	139.6	
12	170.2	
13	119.6	5.53, bd; 3.5 6.27, d, 3.5
14	58.7	4.23, d, 12.3 3.79, d, 12.1
15	16.8	1.61, d; 1.3

s: singlet; m: multiplet; bs: broad singlet; d: doublet; dd: doublet of doublets; ddd: doublet of doublets of doublets; bdd: broad doublet of doublets; ov: (partially) overlapped signals, eq: equatorial, ax: axial.* ^13^C chemical shifts were indirectly determined by GHSQC and GHMBC due to low sample amount.

We further examined whether other costunolide derivatives, such as parthenolide, can be oxidized by CYP71AX36, by additionally expressing parthenolide synthase (*TpPTS*). Expression of CYP71AX36 in the yeast strain endogenously producing parthenolide [EPY300-Leu2d-GAS/CR/GAO/LsCOS|Ura- TpPTS/**CYP71AX36**]resulted in a new peak in the extracted ion chromatogram (EIC) of (C_15_H_21_O_4_) [M + H]^+^  = 265, corresponding to the mass of 14-hydroxyparthenolide at 4.86 min (Supplementary Fig. S11). The peak intensity was very low and did not allow for the purification of sufficient enzyme product for NMR analysis. For the MS^2^ spectrum however, retention time and exact mass (ΔRT < 0.00 min, ΔM = −0.6 ppm) were in good accordance with the reference compound 14-hydroxyparthenolide (Supplementary Fig. S11). When the enzyme was offered germacrene A acid or 8β-hydroxygermacrene A acid in the yeast expression system, no new enzyme product was detected (Supplementary Table S1), suggesting that the enzyme requires a lactone ring in the substrate to catalyze a hydroxylation reaction at position C14. Kinetic studies with wild type and modified CYP71AX36 may lead to a deeper understanding of the enzyme specificity in the future.

### CYP71AX36 converts costunolide to 14-hydroxycostunolide in nicotiana benthamiana

**Figure 4:**
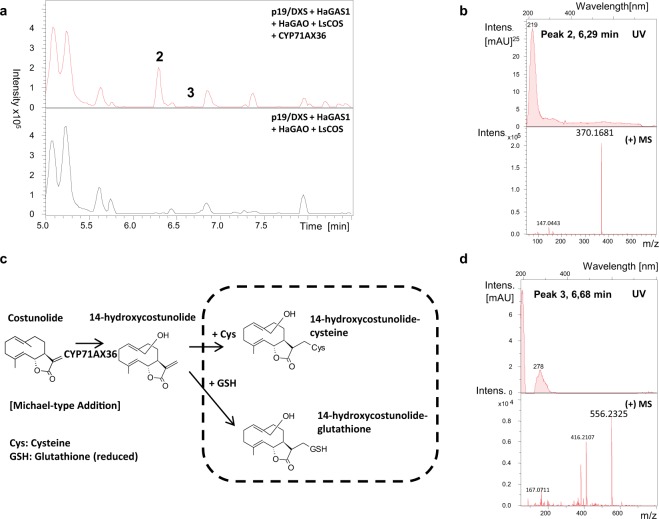
CYP71AX36 catalyzes the 14-hydroxylation of costunolide *in planta*. Comparison of extracts of transformed *N*. *benthamiana* leaves carrying the gene combination [p19/DXS + HaGAS1 + HaGAO + LsCOS]; (n = 3) to [p19/DXS + HaGAS1 + HaGAO + LsCOS + CYP71AX36 (CYP71AX36)]; (n = 3). (**a**) LC-MS chromatograms BPC. (**b**) UV-Vis and MS-spectrum of peak a, (**c**) scheme, 14-hydroxylation of costunolide by CYP71AX36 and subsequent formation of the SH-adducts 14-hydroxycostunolide-cysteine and 14-hydroxycostunolide-glutathione, (**d**) UV-Vis and MS-spectrum of peak (**b**).

Heterologously expressed CYP enzymes have previously shown different properties in different expression hosts^3^. Thus, to verify the activities of the above-mentioned enzymes, we expressed the corresponding cDNAs in tobacco, *Nicotiana benthamiana*. Similar to the yeast system, none of the candidate P450s except for CYP71AX36 accepted three offered substrates, germacrene A acid, 8β-hydroxygermacrene A acid or costunolide. The targeted cDNAs were expressed in *N*. *benthamiana* leaves together with other cDNAs necessary to produce costunolide. Expression was achieved by infiltrating *A*. *tumefaciens* transformed with the plasmids carrying p19/DXS, HaGAS1, HaGAO, LsCOS, and CYP71AX36. Extracts of tobacco leaves transiently transformed with the different cDNA combination described above showed a new peak in the LC-MS chromatogram compared to the control (Fig. 4a, peak **2**, 6.29 min Peak **2** had a mass of [M + H]^+^ = 370.1, corresponding to the mass of a hydroxylated costunolide-cysteine adduct (C_18_H_27_NO_4_S) (Fig. 4b). The formation of SH adducts is commonly known for the production of α-methylene-γ-lactone STLs in *Nicotiana benthamiana*^2,3,12^. If CYP71AX36 carried out the same reaction in plant cells, peak **2** would correspond to 14-hydroxycostunolide-cysteine (Fig. 4c).

An extracted ion chromatogram for the mass of 14-hydroxycostunolide-glutathione (C_25_H_37_N_3_O_8_S) [M + H]^+^ = 556.2 showed another new peak compared to the control (Fig. 4a, peak 3 at 6.68 min, and Supplementary Fig. S9). Indeed, a comparison of the enzyme products to the synthetic SH adducts proved their identity as 14-hydroxycostunolide-cysteine (peak 1, ΔRT < 0.01 min, ΔM = + 0.5 ppm) and 14-hydroxycostunolide-glutathione (peak 2, ΔRT < 0.01 min, ΔM = + 0.0 ppm) (Supplementary Figs S8–S10), based on exact mass, mass spectrum, and retention time comparison. While the amount of 14-hydroxycostunolide-cysteine was higher than that of 14-hydroxycostunolide-glutathione, unconjugated 14-hydroxycostunolide was not detected in transiently transformed *Nicotiana* extracts (Supplementary Table S2). Similar to the expression in yeast, CYP71AX36 did not convert germacrene A acid or 8β-hydroxygermacrene A acid. Collecting all yeast and tobacco data together, we concluded that the enzyme CYP71AX36 is a 14-hydroxylase, and CPY71AX36 was submitted to the GenBank with the accession number MG765530.1.

### Phylogenetic analysis

**Figure 5:**
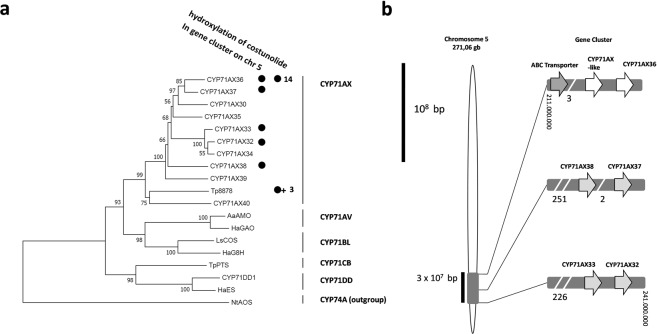
Phylogeny and CYP71AX subfamily gene cluster. (**a**) Phylogeny with cytochrome P450 enzymes involved in sesquiterpenoid metabolism of different Asteraceae plants, calculated from amino acid sequence using neighbor joining method. Bootstrap values calculated from 1,000 iterations are indicated at the nodes. CYP71AX subfamily genes are categorized by their location in the gene cluster and their ability to hydroxylate costunolide. 3,14: positions at which costunolide is hydroxylated, +: enzyme can also use parthenolide as a substrate. (**b**) Gene cluster on *Helianthus annuus* chromosome 5 with six CYP71AX sequences and a putative terpenoid transporter.

The comparison of the amino acid sequences of CYP71AX36 with other P450 enzymes involved in sesquiterpene metabolism identified the putative structural domains and the substrate recognition sites (SRS) of the enzymes, as previously defined for LsCOS and HaG8H^9^ (Supplementary Fig. S12). CYP71AX36 shares 53% amino acid identity with Tp8878, a costunolide and parthenolide 3β-hydroxylase from *Tanacetum parthenium*^2^. Both costunolide-hydroxylating enzymes show a high degree of amino acid similarity in their SRS regions, but CYP71AX36 possesses a 4 amino acid deletion at the upstream of SRS6 that appears to be unique among the enzymes thus far known to be involved in STL metabolism (Supplementary Fig. S12). Many CYP71 P450 genes expressed in capitate glandular trichomes belong to the CYP71AX subfamily, and the costunolide/parthenolide 3β-hydroxylase from *Tanacetum* clusters with other P450 members of this family in the phylogenetic analysis (Fig. 5a, Supplemental Data 2). One of the closest homologs to CYP71AX36 is CYP71AX30 from *Xanthium strumarium*, which was recently considered as a candidate for the involvement in STL metabolism based on gene expression studies^13^. A search for sub-genomic localization revealed that all enzymes thus far characterized in the STL metabolism of *Helianthus annuus*, are scattered across the genome^3^ (Supplementary Fig. S13). However, CYP71AX36 is located as a gene cluster on chromosome five where a number of other P450 genes are co-localized. The specific CYPs are the members of the CYP71AX subfamily and they were all amplified from cDNA of glandular trichomes^3,9^ (Fig. 5b).

### Role of CYP71AX36 in *helianthus*

Despite the diversity of STL in the *Helianthus* genus^14–16^, there is no report of 14-hydroxycostunolide in the *Helianthus* genus, while 8β,14-dihydroxycostunolide (desacetylovatifoline) has been reported from several species of this genus^5,17–20^. The only currently known natural source of 14-hydroxycostunolide is the Asteraceae plant *Clibadium surinamense*^4,11^. Remarkably, 8ß-hydroxy STLs thus far reported from *Helianthus* are normally esterified and have additional modifications on the core skeleton with two exceptions: 9β-hydroxycostunolide (haageanolide) (**5**)^21^ and 8β,14-dihydroxycostunolide (**7**)^5,16–19^. The above findings could be explained as follows: 1. CYP71AX36 can, similar to Tp8878, use hydroxylated versions of costunolide as a substrate. 2. Eupatolide, the precursor of almost all STLs in capitate glandular trichomes of sunflower, can be used as a substrate to generate 8β,14-dihydroxycostunolide. With the discovery of the eupatolide biosynthetic pathway^2,3^, it will be possible to test this hypothesis and to explore the plasticity of the CYP71AX36 enzyme in future studies. This appears to be a promising approach as cytochrome P450 enzymes involved in sesquiterpenoid biosynthetic pathways often show a high substrate plasticity^2,10,22–24^. As our results show, the non-lactonized substrates germacrene A acid and 8β-hydroxygermacrene A acid are not used as a substrate by CYP71AX36. However, similar to the 3β-hydroxylase from *Tanacetum*^2^, the CYP71AX36 can accept other lactonized substrates such as parthenolide and may also accept eupatolide. Testing eupatolide as a substrate would require the generation of a yeast strain that can express functional HaES, which previous studies have not yet accomplished^3,9^.

### Perspectives

In our study, we present the first full ^13^C NMR assignment of 14-hydroxycostunolide. The hydroxylation of the costunolide backbone at position C14 appears to more significantly increase hydrophilicity than the hydroxylation at the C10 ring, given the relatively strong shift in retention time. The C14-hydroxylation of the costunolide backbone is a crucial step to produce several STL derivatives of pharmaceutical interest. CYP71AX36 accepts parthenolide as a substrate, as it is the case with the structurally similar 3β-hydroxylase from *Tanacetum* Tp8878. If the production of 14-hydroxyparthenolide can be improved, it might be possible to directly produce the promising antileukemic drug 14-hydroxyparthenolide^7,25,26^ (**10**) and its derivatives from simple sugar units in yeast culture^2^. Introduction of the 14-hydroxy-group to eupatolide (8β-hydroxycostunolide) would lead to desacetylovatifoline that has shown activity against a set of cancer cell lines^5^. A chemical oxidation could convert 14-hydroxycostunolide to taraxinic acid that has been shown to be active against leukemia stem cells^6^. In addition to the α-methylene-γ-lactone, α,β-unsaturated alcohol groups such as the 14-hydroxy alcohol group in combination with the 1–10 double-bond can contribute to the bioactivity of STL^4,27^ which would in part explain the bioactivity of 14-hydroxylated costunolide derivatives.

## Experimental Section

### Nucleic acid extraction and PCR

Candidate P450 gene search, RNA and gDNA extraction, cDNA synthesis and RACE PCR were carried out as described previously^3,9,28^. The analysis of CYP71AX36 gene expression by qPCR was tested. However, as many very similar gene sequences were expressed in the same tissue, sequencing of the very short qPCR amplicons could not unambiguously corroborate their identity as CYP71AX36 transcripts. Hence, CYP71AX36 expression was analyzed by PCR amplifying the whole open reading frame of CYP71AX36 from cDNA (primers 1–2, Supplementary Table S4). As a control the housekeeping gene ubiquitin was amplified using primers 3–4. PCR was carried out using Phusion polymerase (Phusion, Finnzymes) according to the manufacturer’s instructions with 1 ng cDNA and 0.5 µM primers in a volume of 40 µl. The program for the amplification of CYP71AX36 and ubiquitin was: 2:00 min; 98 °C, 39 cycles of 0:30 min 98 °C, 0:30 min 56 °C, 1:30 min 72 °C and 72 °C 2:00. PCR products were cloned into blunt end vector pSC-A (Agilent) and sequenced using primers 5–6.

### Enzyme characterization in yeast and *nicotiana benthamiana*

Candidate cytochrome P450 genes were amplified from capitate glandular trichome cDNA with a proofreading polymerase (Phusion) introducing restriction sites suitable for subsequent cloning and subcloned in pJet1.2 blunt vector according to the manufacturer’s instructions. Subsequently, CYP71AX39 was cloned into the EcoRI and ClaI restriction sites (primers 7–8) and all other candidate genes into the EcoRI and SpeI restriction sites (primers 9–24, Supplementary Table S4) of the multiple cloning site 1 of pESC-Ura expression vectors (candidate vectors) as previously described^9^. Using primers (25–26, Supplementary Table S4), TpPTS was PCR amplified from pBinPlus-TpPTS^2^ thereby inserting BamHI and HindIII restriction sites. The purified PCR product was subcloned into the pJet2.1 blunt vector (Thermo Fisher Scientific). The subcloned TpPTS gene was then cut out of the vector pJet2.1–TpPTS using BamHI and HindIII. The vectors pESC-Ura and pESC-Ura-CYP71AX36 were digested with BamHI and HindIII, and additionally with SrfI to reduce the background of religated vector. All restriction enzymes were purchased from New England Biolabs and restriction digestion was carried out according to the manufacturers’ instructions. The TpPTS fragment was then ligated into the free multiple cloning site 2 of pESC-Ura and pESC-Ura-CYP71AX36 using T4 DNA Ligase (Thermo Fisher Scientific) according to manufacturer’s instructions. The previously generated vectors Leu2d-GAS/GAO/CR, Leu2d-GAS/GAO/CR/HaG8H and Leu2d-GAS/GAO/CR/LsCOS^9,10,29^ were used as substrate vectors. The FPP overproducing yeast strain EPY300 was subsequently transformed with a combination of a substrate and a candidate vector by heat-shock transformation^30,31^. USER cloning of enzyme genes into the *Agrobacterium* vector was transformed as described previously^3,32^. The genes up-stream in the STL pathway transiently transformed into *Nicotiana benthamiana* were CfDXS, HaGAS1, HaGAO, and LsCOS as previously described^3^. All vector constructs were sequenced using primers flanking the multiple cloning sites (primers 27–36). Enzyme characterization in yeast was carried out as previously described^9,10^ and extracts of yeast cultures were analyzed on a HPLC-DAD reversed phase chromatography. For NMR analysis 14-hydroxycostunolide was purified from a 0.5 L culture of yeast strain EPY300-Leu2d-GAS/GAO/CR/LsCOS| Ura-CYP71AX36 grown for five days in 2 L Erlenmeyer flasks in synthetic complete dropout medium using 0.1 M MOPS buffer to maintain the pH at 6.5^12^. The sterile filtered yeast culture was extracted three times with ethyl acetate and the dried extract was redissolved for preparative HPLC. Thirty fractions of the 14-hydroxy-costunolide peak at 5.5 min were collected and the dried pellet redissolved for NMR spectroscopy in CDCl_3_. *Agrobacterium* mediated pathway engeneering was performed in *Nicotiana benthamiana* via strain AGL1 according to a previously established standard procedure^3,32,33^.

### NMR analysis

NMR analysis of the isolated compound was carried out using a 500 MHz Varian Unity Inova (Varian, Inc./Agilent Technologies) spectrometer. The ^1^H and ^13^C NMR chemical shifts were referenced to the residual solvent signals at δ_H/C_ 7.26/77 ppm relative to TMS.1D and 2D homonuclear NMR spectra were measured with standard Varian pulse sequences. Adiabatic GHSQCAD, GHMBCAD and ROESYAD spectra were recorded using CHEMPACK 7.2 pulse sequences (implemented in Varian Vnmrj 4.2 spectrometer software). Spectra were either processed with Vnmrj 4.2 or Spinworks 4.2.8 (Copyright 2017, K. Marat, University of Manitoba, Canada) The structure of 14-hydroxycostunolide was unambiguously identified by evaluation of all NMR spectra. Modeling of 14-hydroxycostunolide as a comparison to NMR results was performed with Chem3D Pro (Version 10, Cambridge Soft).

### HPLC-DAD and LC-MS analysis

Extraction of transiently transformed *Nicotiana benthamiana* plants in methanol and yeast cultures with ethyl acetate was performed as previously described^3,9^. HPLC-DAD screening of yeast culture extracts and LC-MS analysis of extracts from transiently transformed *Nicotiana benthamiana* plants as well as synthetic adducts was performed as previously described^3,10^. LC-MS parameters for yeast enzyme analysis and comparison of synthetic adducts were: Agilent 1290 UHPLC with an Acquity CSHC18 column (1.7 um particle size, 150 mm × 2.1 mm) combined with a Q Exactive Plus (ThermoFisher Scientific) mass spectrometer. Solvent program: 10 min 10–70% B, 5 min 70–90% B, 1 min 90–10% B, 1 min 10% B; A: ddH_2_O/0.1% (v/v) FA, B: ACN/0.1%(v/v) FA; A/B: LC-MS-Grade; 0.3 ml/min, column temperature.: 40 °C. Q Exactive Plus mass spectrometer equipped with HESI-II probe. Polarity mode: Positive, Scan Mode: data-dependent MS/MS. Mass Range: 140–1200 amu for Full-MS. Ion source settings: Spray Voltage = 4.2 KV, Vaporizer = 380 °C, Ion Transfer Tube = 360 °C, S-Lens = 50%, Sheath Gas = 60, Auxiliary gas = 20, Resolution MS = 70,000 (FWHM at m/z 200), Resolution MS2 = 17,500 (FWHM at m/z 200), Stepped Collision Energy (NCE) = 20–30–40. Costunolide and parthenolide were purchased from a commercial supplier (Selleck Chemicals). The reference compound 14-hydroxycostunolide was provided by Dr. Rudi Fasan (University of Rochester). Cysteine and glutathione adducts of costunolide and 14-hydroxycostunolide were synthesized as previously described^12^.

### Subgenomic localization, sequence alignment and phylogeny

The subgenomic localization of cytochrome P450 enzymes was analyzed with the genome browser tool provided by the compositae genome project (www.sunflowergenome.org). Amino acid sequences of candidate genes and characterized P450 enzymes were aligned using BioEdit (Version 7.2.5, IbisBiosciences) and a phylogeny was calculated with the neighbor-joining method with 1000 bootstraps in MEGA6.

## Supplementary information

Supplementary Dataset 2

Supporting Information: Characterization of CYP71AX36 from Sunflower (Helianthus annuus L., Asteraceae)
