# Supplementary material for: Characterization of CYP71AX36 from Sunflower (Helianthus annuus L., Asteraceae)
Source: Sci Rep. 2019 Oct 4;9:14295. doi: 10.1038/s41598-019-50520-6 (PMC6778120; doi:10.1038/s41598-019-50520-6)
Supplement: Supplementary file 2 — Supporting Information: Characterization of CYP71AX36 from Sunflower (Helianthus annuus L., Asteraceae) [file 41598_2019_50520_MOESM2_ESM.docx]

**Supporting Information**

**Characterization of CYP71AX36 from Sunflower (*Helianthus annuus* L., Asteraceae)**

**Maximilian Frey^a*^, Iris Klaiber^b^, Jürgen Conrad^c^, Aylin Bersch^a^, Irini Pateraki^d^, Dae-Kyun Ro^e^, Otmar Spring^a^**

^a^ Institute of Botany, University of Hohenheim, Garbenstraße 30, 70593 Stuttgart, Germany

^b^ Mass Spectrometry Unit, Core Facility Hohenheim, University of Hohenheim, Emil-Wolff-Str. 12, 70599 Stuttgart, Germany

^c^ Institute of Chemistry, University of Hohenheim, Garbenstraße 30, 70593 Stuttgart, Germany

^d^ Department of Plant and Environment al Sciences, Faculty of Science, University of Copenhagen, Thorvaldsensvej 40, Frederiksberg C, Denmark

^e^ Department of Biological Sciences, University of Calgary, Calgary T2N 1N4, Canada

**Supplementary Information Table of Contents**

**Content Pages**

Supplementary Table S1: Control experiments in yeast 3S

Supplementary Table S2: Metabolites detected in extracts of transiently transformed *N. benthamiana* leaves 4S

Supplementary Table S3: Genes heterologously expressed in yeast and *N. benthamiana 5S*

Supplementary Table S4: Oligonucleotides used in this work 6S

Supplementary Figure S1: Scheme for the co-expression of enzymes to produce 14-hydroxycostunolide in yeast 7S

Supplementary Figure S2: Modeling of the stereochemistry of 14-hydroxycostunolide 8S

Supplementary Figure S3: 1H NMR spectrum of 14-hydroxycostunolide in CDCl_3_ at 500 MHz 9S

Supplementary Figure S4: gCOSY NMR spectrum of 14-hydroxycostunolide in CDCl_3_ at 500 MHz 10S

Supplementary Figure S5: ROESYAD NMR spectrum of 14-hydroxycostunolide in CDCl_3_ at 500 MH 11S

Supplementary Figure S6: gHSQCAD NMR spectrum of 14-hydroxycostunolide in CDCl_3_ at 500 MHz 12S

Supplementary Figure S7: gHMBCAD NMR spectrum of 14-hydroxycostunolide in CDCl_3_ at 500 MHz 13S

Supplementary Figure S8: Comparison of CYP71AX36 enzyme products to synthetic cysteine adducts 14S

Supplementary Figure S9: EIC showing the *in planta* production of 14-hydroxycostunolide-glutathione 15S

Supplementary Figure S10: Comparison of CYP71AX36 enzyme products to synthetic glutathione adducts 16S

Supplementary Figure S11: Expression of CYP71AX36 downstream of TpPTS and comparison to 14-hydroxyparthenolide 17S

Supplementary Figure S12: Amino acid alignment of enzymes involved in sesquiterpene lactone biosynthesis 18S

Supplementary Figure S13: Localization of STL pathway enzymes on the chromosomes of *Helianthus annuus* 19S

References 20S

Supplementary Table S1: Control experiments in yeast

| Experiment | New product compared to control |
| --- | --- |
| **Yeast EPY 300 wild type** | **Wild type control** |
| GAS + GAO (4n) | Germacrene A acid |
| GAS + GAO + HaG8H (4n) | 8β-hydroxygermacrene A acid |
| GAS + GAO + LsCOS (4n) | Costunolide |
| GAS + GAO + CYP71AX36 (4n) | None |
| GAS + GAO + LsCOS +CYP71AX36 (4n) | 14-hydroxycostunolide |
| GAS + GAO + HaG8H + CYP71AX36 (4n)  GAS + GAO + LsCOS + TpPTS (3n)  GAS + GAO + LsCOS + TpPTS + CYP71AX36 (6n) | None  Parthenolide  14-hydroxyparthenolide |

The farnesyl pyrophosphate overproducing yeast strain EPY300 was transformed with the substrate vectors pESC-Leu2d-GAS/GAO/CR, pESC-Leu2d-GAS/GAO/CR/HaG8H and pESC-Leu2d-GAS/GAO/CR/LsCOS producing germacrene A acid, 8β-hydroxygermacrene A acid, and costunolide. When the substrate vectors were combined with empty pESC-Ura vectors no new enzyme was produced. Combination of the substrate vectors with pESC-Ura-CYP71AX36 led to no new product downstream of germacrene A acid and 8β-hydroxygermacrene A acid, but showed conversion of costunolide to 14-hydroxycostunolide.

Supplementary Table S2: Metabolites detected in extracts of transiently transformed *N. benthamiana* leaves

|  | p19/DXS+HaGAS1  +HaGAO+LsCOS (3 n) | p19/DXS+HaGAS1  +HaGAO+LsCOS+  CYP71AX36 (3n) ^(a)^ ^(d)^ |
| --- | --- | --- |
| Germacrene A acid | -  ^(b)^ | - |
| Costunolide | **(+)** | **(+)** |
| Costunolide-cysteine | **+++** ^(c)^ | **+++** |
| Costunolide-GSH | **++** | **++** |
| 14-hydroxycostunolide | - | ***** |
| 14-hydroxycostunolide-cysteine | - | **+++** |
| 14-hydroxycostunolide-GSH | - | **+** |

^(a)^ HaGAS1: *Helianthus annuus* germacrene A synthase 1, HaGAO: *Helianthus annuus* germacrene A oxidase, LsCOS: *Lactuca sativa* costunolide synthase

^(b)^ -: not detected by LC-MS.

^(c)^ **+** to **+++**: relative amount of detected metabolite, **(+)** traces detected, * presumed direct product, not detected.

^(d)^ When CYP71AX36 was expressed downstream of HaGAO or HaG8H neither of the costunolide or 14-hydroxycostunolide derivatives could be detected.

Supplementary Table S3: Genes heterologously expressed in yeast and *N. benthamiana*

| Experiment | Name | Organism | Accession nr. | Reference |
| --- | --- | --- | --- | --- |
| Pathway reconstruction in yeast | AaCR | *Artemisia annua* | DQ984181 | ^1^ |
|  | LsGAS2 | *Lactuca sativa* | AF489965 | ^1^ |
|  | LsGAO | *Lactuca sativa* | GU198171 | ^1^ |
| Pathway reconstruction in tobacco | HaGAS1 | *Helianthus annuus* | DQ016667 | ^2^ |
|  | HaGAO | *Helianthus annuus* | GU256646 | ^1^ |
|  | CfDXS | *Coleus forskohlii* | KP889115 | ^3^ |
| Pathway reconstruction in yeast and tobacco | LsCOS | *Lactuca sativa* | HQ439599 | ^4^ |
|  | HaG8H | *Helianthus annuus* | HQ439590 | ^4^ |
| Re-evaluated enzyme- | CYP71AX40 | *Helianthus annuus* | HQ439594 | ^4^, candidate S1 |
| candidates ^4,5^ | CYP71AX37 | *Helianthus annuus* | HQ439598 | ^4^, candidate S3 |
|  | CYP71AX32 | *Helianthus annuus* | HQ439593 | ^4^, candidate, C28 |
|  | CYP71AX38 | *Helianthus annuus* | HQ439592 | ^4^, candidate C100 |
|  | CYP71AX33 | *Helianthus annuus* | HQ439593 | ^5^, candidate C28B |
|  | CYP71AX35 | *Helianthus annuus* | MG765528 | ^5^, candidate M22A |
|  | CYP71AX39 | *Helianthus annuus* | MG765529 | ^5^, candidate M22A |
|  | CYP71AX36 | *Helianthus annuus* | MG765530 | ^5^, candidate M22A |

Supplementary Table S4: Oligonucleotides used in this work

| No. | Oligonucleotide | Sequence 5´-3´ |
| --- | --- | --- |
| 1 | CYP71AX36_F | ATGTCTTTCAACCTACAAGTTTTTCTC |
| 2 | CYP71AX36_R | TCACATGGAGTCACCGAGAAA |
| 3 | HexaUbiQ_F ^6^ | CTGTTTCGCTTCGTCTCTTTCA |
| 4 | HexaUbiQ_F^6^ | CTGAGCCTGAGCACGATGA |
| 5 | t3 | GCAATTAACCCTCACTAAAGG |
| 6 | t7 | TAATACGACTCACTATAGGG |
| 7 | CYP71AX39_F_EcoRI | GTCAATGAATTCATGGGGAGATTTAGCAAGTCTATCAAT |
| 8 | CYP71AX39_R_ClaI | *GTCAAT*ATCGAT**TTA**AGCAATAGGAGTTGCTGAAACTAGTATAG |
| 9 | CYP71AX32/33_EcoRI | *GTCAAT*GAATTC**ATG**TCTTTCAACCTACAAGTT |
| 10 | CYP71AX32/33_SpeI | *GTCAAT*ACTAGT**TTA**CTCCCTAGGAGTTGCTTTGAC |
| 11 | CYP71AX34_F_EcoRI | *GTCAAT*GAATTC**ATG**TCTTTCAACCTACAAGTTTTTGTCTTC |
| 12 | CYP71AX34_R_SpeI | *GTCAAT*ACTAGT**CTA**CTCCCTAGGAGTTGCTTTGACAAG |
| 13 | CYP71AX35_F_EcoRI | *GTCAAT*GAATTC**ATG**TCTTTCACTTTGCAAATTGTTCTC |
| 14 | CYP71AX35_R_SpeI | *GTCAAT*ACTAGT**TTA**TTACTTATAAGAAGTTGCTGTAACAAGTATAGG |
| 15 | CYP71AX36_F_EcoRI | *GTCAAT*GAATTC**ATG**TCTTTCAACCTACAAGTTTTTCTC |
| 16 | CYP71AX36_R_SpeI | *GTCAAT*ACTAGT**TCA**CATGGAGTCACCGAGAAA |
| 17 | CYP71AX37_F_EcoRI | *GTCAAT*GAATTC**ATG**TCTTTGGACTTTCAAGTT |
| 18 | CYP71AX37_R_SpeI | *GTCAAT*ACTAGT**TTA**CTCGCAAGGAGTTGGTATGAC |
| 19 | CYP71AX38_EcoRI | *GTCAAT*GAATTC**ATG**TCTTTCAACTTGCAAGTTTTTCTCCTCTC |
| 20 | CYP71AX38_SpeI | *GTCAAT*ACTAGT**CTA**CTCACAAGGAGTTGCAACAACAC |
| 21 | CYP71AX40_F_EcoRI | *GTCAAT*GAATTC**ATG**GAAATTTTTCCATCTTTCCA |
| 22 | CYP71AX40_R_SpeI | *GTCAAT*ACTAGT**CTA**GACCCTAGGAGTAGCAACAACTAG |
| 23 | CYP71DD6_F_EcoRI | *GTCAAT*GAATTC**ATG**GATTTCTTGACATACTTGCCA |
| 24 | CYP71DD6_R_SpeI | *GTCAAT*ACTAGT**CTA**AGCTTGTGTATTGTGCTTGATGGG |
| 25 | TpPTS_F_BamHI | *GTCAAT*GGATCC**ATG**GATACCTCTACAAGTTTTCCTTCG |
| 26 | TpPTS_R_HindIII | *GTCAAT*AAGCTT**CTA**GATGTGCTTGATGGGAATAAGGA |
| 27 | pJet1.2_F | CGACTCACTATAGGGAGAGCGGC |
| 28 | pJet1.2_R | AAGAACATCGATTTTCCATGGCAG |
| 29 | Gal1-F | ATTACTTCTTATTCAAATGTA |
| 30 | Gal1-R | AATATAAATAACGTTCTTAA |
| 31 | Gal10-F | GGATATGTATATGGTGGTAATG |
| 32 | Gal10-R | GACAACCTTGATTGGAGAC |
| 33 | M13F | GTAAAACGACGGCCAGT |
| 34 | M13R | GCGGATAACAATTTCACACAGG |
| 35 | USERseq_F | AGAGGACGACCTGCAGGC |
| 36 | USERseq_R | GCATGGGTCGACGAGC |

Supplementary Figure S1: Scheme for the co-expression of enzymes to produce 14-hydroxycostunolide in yeast

The scheme shows the co-expression of five genes involved in sesquiterpene lactone metabolism to produce 14-hydroxycostunolide in yeast. The FPP overproducing yeast strain EPY300 has been transformed with five genes encoded on two vectors. In three reactions FPP is converted by the enzymes encoded on the vector Leu2d (substrate vector): 1. LsGAS2 converts FPP to germacrene A, 2. LsGAO and AaCR convert germacrene A to germacrene A acid, 3. LsCOS and AaCR convert germacrene A acid to costunolide. The reaction from costunolide to 14-hydroxycostunolide is carried out by CYP71AX36 from the pESC-Ura vector (candidate vector) and the AaCR from the pESC-Leu2d vector. The end product 14-hydroxycostunolide is transported out of the yeast cells and accumulates in the yeast culture medium from where it is subsequently extracted. (Vectors are simplified and reduced to the encoded STL pathway genes).

Supplementary Figure S2. Modeling of the stereochemistry of 14-hydroxycostunolide

Supplementary Figure S3. 1H NMR spectrum of 14-hydroxycostunolide in CDCl_3_ at 500 MHz.

Supplementary Figure S4. gCOSY NMR spectrum of 14-hydroxycostunolide in CDCl_3_ at 500 MHz.

Supplementary Figure S5. ROESYAD NMR spectrum of 14-hydroxycostunolide in CDCl_3_ at 500 MHz.

Supplementary Figure S6. gHSQCAD NMR spectrum of 14-hydroxycostunolide in CDCl_3_ at 500 MHz.

Supplementary Figure S7. gHMBCAD NMR spectrum of 14-hydroxycostunolide in CDCl_3_ at 500 MHz.

Supplementary Figure S8: Comparison of CYP71AX36 enzyme products to synthetic cysteine adducts

Comparison of extracts from *N. benthamiana* leaves (a) [p19/DXS + HaGAS1 + HaGAO + LsCOS + CYP71AX36] to (b) synthetic 14-hydroxycostunolide-cysteine. **a**, EIC (C_18_H_27_NO_5_S) [M+H]^+^ =370. **b**, (+) MS-spectra at 3.93 min. **c**, (+) MS-MS-spectra at 5.56 min. * unspecific peak

Supplementary Figure S9: EIC showing the *in planta* production of 14-hydroxycostunolide-glutathione

Comparison of extracts of transformed *N. benthamiana* leaves carrying the gene combination [p19/DXS + HaGAS1 + HaGAO + LsCOS] (black); (n = 3) to [p19/DXS + HaGAS1 + HaGAO + LsCOS + CYP71AX36] (n = 3) (red); EIC (C_25_H_37_N_3_O_9_S) [M+H]^+^ =556. Note that the scale is approximately 50x lower than in the TIC chromatogram shown in figure 4 A.

Supplementary Figure S10: Comparison of CYP71AX36 enzyme products to synthetic glutathione adducts

Comparison of extracts from *N. benthamiana* leaves [p19/DXS + HaGAS1 + HaGAO + LsCOS+ CYP71AX36] (black) to synthetic 14-hydroxyostunolide-glutathione (red). **a**, EIC (C_25_H_37_N_3_O_9_S) [M+H]^+^ =556: **b**, (+) MS-spectra at 4.209 min. * unspecific peak

Supplementary Figure S11: Expression of CYP71AX36 downstream of TpPTS and comparison to 14-hydroxyparthenolide

LC-MS comparison of reference compound 14-hydroxyparthenolide (black) to extracts from yeast strains [EPY300-Leu2d-GAS/CR/GAO/LsCOS | Ura- TpPTS/**CYP71AX36**] (red) and negative control [EPY300-Leu2d-GAS/CR/GAO/LsCOS | Ura- TpPTS] (green); a, EIC (C15H21O4) [M+H]^+^ =265: b, (+) MS-spectra at 4.86 min.

Supplementary Figure S12: Amino acid alignment of enzymes involved in sesquiterpene lactone biosynthesis

Amino acid sequence alignment, SRS: substrate recognition site, structural features in italics: TMD: trans-membrane domain, FxPERF and FxxGxRxCxG motif. *: amino acid deletion characteristic to CYP71AX subfamily. Dark grey shading: identical amino acid, light grey shading: similar amino acid.

Supplementary Figure S13: Localization of STL pathway enzymes on the chromosomes of *Helianthus annuus*

The 17 chromosomes of the sunflower genome and the chromosome size in gigabases are shown. The positions of terpene synthases, and those of published and new cytochrome P450 genes are indicated. Grey shading: gene cluster region on chromosome 5, genes that exist in more than one copy are indicated by roman numbers (I-II). Simplified scheme from www.sunflowergenome.org, HA412 bronze assembly. Sesquiterpene synthases, localized on the chromosomes: FPS (farnesyl pyrophosphate synthase), GAS1 (germacrene A synthase 1), GAS2 (germacrene A synthase 2), CS (cadinene synthase); Cytochrome P450 enzymes und enzyme candidates, localized on chromosomes: GAO (germacrene A synthase), HaG8H (germacrene A acid 8β-hydroxylase), and HaES (eupatolide synthase), enzyme candidates CYP71AX32, CYP71AX33, CYP71AX36, CYP71AX37, CYP71AX38, CYP71AX40, no unambiguous localization on the chromosome was possible for: HaGAS3 (germacrene A synthase 3), bisabolene synthases K7 und K11, cytochrome P450 candidates CYP71AX34, CYP71AX35, CYP71AX39.

References

1. Nguyen, D. T. *et al.* Biochemical conservation and evolution of germacrene A oxidase in asteraceae. *J. Biol. Chem.* **285,** 16588–16598 (2010).

2. Göpfert, J. C., Macnevin, G., Ro, D.-K. & Spring, O. Identification, functional characterization and developmental regulation of sesquiterpene synthases from sunflower capitate glandular trichomes. *BMC Plant Biol.* **9,** 86 (2009).

3. Luo, D. *et al.* Oxidation and cyclization of casbene in the biosynthesis of *Euphorbia* factors from mature seeds of *Euphorbia lathyris* L. *Proc. Natl. Acad. Sci.* 201607504 (2016). doi:10.1073/pnas.1607504113

4. Ikezawa, N. *et al.* Lettuce costunolide synthase (CYP71BL2) and its homolog (CYP71BL1) from sunflower catalyze distinct regio- and stereoselective hydroxylations in sesquiterpene lactone metabolism. *J. Biol. Chem.* (2011). doi:10.1074/jbc.M110.216804

5. Frey, M., Schmauder, K., Pateraki, I. & Spring, O. Biosynthesis of Eupatolide—A Metabolic Route for Sesquiterpene Lactone Formation Involving the P450 Enzyme CYP71DD6. *ACS Chem. Biol.* **13,** 1536–1543 (2018).

6. Grasse, W., Zipper, R., Totska, M. & Spring, O. Plasmopara halstedii virus causes hypovirulence in Plasmopara halstedii, the downy mildew pathogen of the sunflower. *Fungal Genet. Biol.* **57,** 42–47 (2013).
